# Heritability of the glycan clock of biological age

**DOI:** 10.3389/fcell.2022.982609

**Published:** 2022-12-22

**Authors:** Anika Mijakovac, Azra Frkatović, Maja Hanić, Jelena Ivok, Marina Martinić Kavur, Maja Pučić-Baković, Tim Spector, Vlatka Zoldoš, Massimo Mangino, Gordan Lauc

**Affiliations:** ^1^ Division of Molecular Biology, Department of Biology, Faculty of Science, University of Zagreb, Zagreb, Croatia; ^2^ Genos Glycoscience Research Laboratory, Zagreb, Croatia; ^3^ Department of Twin Research and Genetic Epidemiology, King’s College London, London, United Kingdom; ^4^ NIHR Biomedical Research Centre at Guy’s and St Thoma’s Foundation Trust, London, United Kingdom; ^5^ Department of Biochemistry and Molecular Biology, Faculty of Pharmacy and Biochemistry, University of Zagreb, Zagreb, Croatia

**Keywords:** heritability, glycan clock, aging biomarker, biological age, IgG glycosylation

## Abstract

Immunoglobulin G is posttranslationally modified by the addition of complex N-glycans affecting its function and mediating inflammation at multiple levels. IgG glycome composition changes with age and health in a predictive pattern, presumably due to inflammaging. As a result, a novel biological aging biomarker, glycan clock of age, was developed. Glycan clock of age is the first of biological aging clocks for which multiple studies showed a possibility of clock reversal even with simple lifestyle interventions. However, none of the previous studies determined to which extent the glycan clock can be turned, and how much is fixed by genetic predisposition. To determine the contribution of genetic and environmental factors to phenotypic variation of the glycan clock, we performed heritability analysis on two TwinsUK female cohorts. IgG glycans from monozygotic and dizygotic twin pairs were analyzed by UHPLC and glycan age was calculated using the glycan clock. In order to determine additive genetic, shared, and unique environmental contributions, a classical twin design was applied. Heritability of the glycan clock was calculated for participants of one cross-sectional and one longitudinal cohort with three time points to assess the reliability of measurements. Heritability estimate for the glycan clock was 39% on average, suggesting a moderate contribution of additive genetic factors (A) to glycan clock variation. Remarkably, heritability estimates remained approximately the same in all time points of the longitudinal study, even though IgG glycome composition changed substantially. Most environmental contributions came from shared environmental factors (C), with unique environmental factors (E) having a minor role. Interestingly, heritability estimates nearly doubled, to an average of 71%, when we included age as a covariant. This intervention also inflated the estimates of unique environmental factors contributing to glycan clock variation. A complex interplay between genetic and environmental factors defines alternative IgG glycosylation during aging and, consequently, dictates the glycan clock’s ticking. Apparently, environmental factors (including lifestyle choices) have a strong impact on the biological age measured with the glycan clock, which additionally clarifies why this aging clock is one of the most potent biomarkers of biological aging.

## Introduction

Glycosylation is a series of enzymatic reactions in which carbohydrates are attached to other molecules (e.g., proteins or lipids) resulting in the formation of complex carbohydrates and glycoconjugates commonly referred to as “glycans.” Protein glycosylation is one of the most frequent secondary modifications (Mariño et al., 2010). The addition of different glycan extensions (alternative glycosylation) greatly affects the structure and function of glycoproteins and it can be compared to changes in protein sequences (Dalziel et al., 2014). The main difference is that genes unquestionably determine the protein sequence, while there is no genetic template for the glycans (Taniguchi et al., 2014). Instead, glycosylation is controlled by many genes and their products, interacting in complex networks that are furthermore influenced by epigenetic modifications and the environment (Freeze, 2006; Abbott et al., 2008; Nairn et al., 2008; Lauc et al., 2014; Klarić et al., 2020). Heritability analysis of plasma glycans revealed that the majority of traits have high heritability estimates, indicating a tight genetic control of glycosylation (Zaytseva et al., 2020). Similar results were observed for glycans attached to immunoglobulin G (IgG), where only a minority of traits exhibited higher environmental contribution (Menni et al., 2013). Glycosylation of IgG antibody is especially interesting as it dramatically affects its function and acts as a molecular switch between pro- and anti-inflammatory immune responses (Gornik et al., 2012; Li et al., 2017; Alter et al., 2018). Aberrant IgG glycosylation is commonly observed in various pathological states, including autoimmune and inflammatory disorders, but the largest change of IgG glycome composition occurs during aging and in age-related conditions (Gudelj et al., 2018).

Aging is defined as the accumulation of molecular, cellular and organ damage over time that leads to loss of function and, consequently, increased vulnerability to disease (Fontana et al., 2010). This age-associated physiological decline is termed biological aging and it separates individuals of the same chronological age based on their health and functionality (Kavur et al., 2021). The accurate prediction of chronological and biological age from biochemical parameters became a priority in the aging field (Mitnitski and Rockwood, 2014; Jylhävä et al., 2017; Porter et al., 2021). It is hypothesized that biological age, influenced by different molecular hallmarks such as telomere shortening, genomic instability and cellular senescence, gives rise to age-related disease risk. Therefore, biological age is a much more potent prognostic tool for health outcomes than chronological age, and more importantly, it can be reversed (Jurić et al., 2020; Greto et al., 2021; Macdonald-Dunlop et al., 2022). Since this notion has been proposed, different predictors of biological age, termed aging clocks, were constructed using various methods (Horvath et al., 2020; Macdonald-Dunlop et al., 2022).

One of the most prominent aging clocks are based on DNA methylation, which is strongly associated with chronological age (Hannum et al., 2013; Horvath, 2013; Porter et al., 2021). Unlike DNA methylation, glycosylation of IgG does not predict chronological age with high accuracy. IgG glycosylation changes with aging in a predictive pattern which lead to the development of the glycan clock, based entirely on N-linked glycans attached to IgG. The glycan clock predicts chronological age with an estimated error of 9.7 years, but its acceleration associates with various biochemical and physiological traits related to inflammation and poor metabolic health reflective of biological aging (Krištić et al., 2014). The high plasticity of IgG glycome in response to environmental stimuli and its unique role in the immune response make the glycan clock one of the best predictors of biological age (Le Couteur et al., 2014; Russell et al., 2018). New studies also showed a possibility of glycan clock reversal with simple lifestyle changes such as weight loss, dietary supplements and exercise (Peng et al., 2019; Tijardović et al., 2019; Greto et al., 2021; Deriš et al., 2022b) as well as medical interventions such as bariatric surgery and regulation of sex hormones (Jurić et al., 2020; Greto et al., 2021).

Nevertheless, no research was done to determine to which extent the glycan clock can be modified and how much it depends on fixed genetic information. To answer this question, we performed a heritability analysis on the glycan clock data from TwinsUK (Verdi et al., 2019). A classical twin design (Visscher, 2004) enabled us to differentiate the contribution of genetic, shared environmental and unique environmental factors to phenotypic variation of the glycan clock. To shed more light on the effect of chronological age on the glycan clock variation, we performed the same analysis including age as a covariate. With this study, we gave an answer to an intriguing question on how much our environment and lifestyle choices influence the biological age measured by the glycan clock and to what proportion genes determine the glycan age.

## Materials and methods

### Study participants

Participants of this study were monozygotic (MZ) and dizygotic (DZ) adult female twins from the TwinsUK cohort. The TwinsUK is a nationwide same-sex twin registry based in the United Kingdom hosted by the Department of Twin Research and Genetic Epidemiology at King’s College London (Verdi et al., 2019). All twins were recruited as volunteers and were not selected by any particular trait. In this study, we included 4,282 female twins for whom the IgG glycome measurements were obtained as a part of a cross-sectional study (Freidin et al., 2016). Among these, 1,598 female twins were later included in a longitudinal study where the IgG glycome was assessed in three time points. The number of male twins was small in both cohorts to allow for accurate estimation of model for the glycan clock calculation so all male participants were excluded from the study.

### Immunoglobulin G isolation and glycan analysis from plasma samples

**FIGURE 1:**
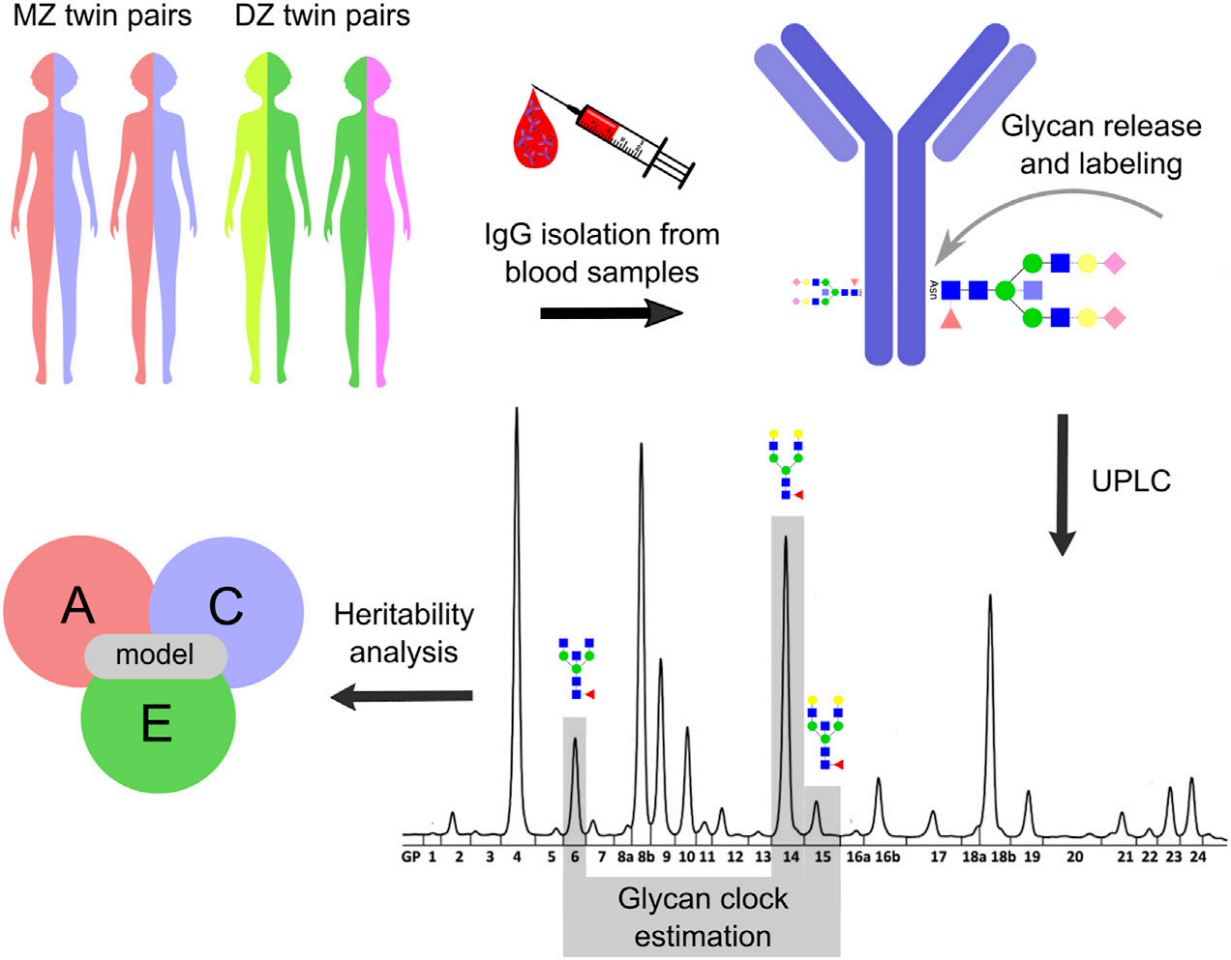
Study design. Heritability analysis was performed on the glycan clock data from the TwinsUK registry. The study included 479 monozygotic (MZ) and 1,193 dizygotic (DZ) female twin pairs. Blood samples were taken from twin pairs involved in one cross-sectional and one longitudinal cohort with three time points. IgG was isolated from collected blood samples which was followed by N-glycan release and labeling. IgG N-glycome composition was analyzed for the cross-sectional cohort by ultra-high-performance liquid chromatography (UHPLC) and the resulting IgG N-glycan chromatograms were separated into 24 distinct peaks. The glycan clock was estimated from GP6, GP14 and GP15 peaks as described in Krištić et al., 2014. Utilizing ACE model the heritability of the glycan clock was calculated. From this model, the contribution of genetic (A), shared environmental (C) and unique environmental (E) factors on the phenotypic variation of the glycan clock was estimated. Note that the IgG N-glycan analysis was slightly different for the longitudinal cohort (described in Materials and methods) but was not included for the simplicity of the display.

IgG was isolated from plasma samples obtained from MZ and DZ twin pairs (Figure 1). IgG isolation procedure was carried out using protein G monolithic plates (BIA Separations, Ajdovščina, Slovenia) as described previously (Pučić et al., 2011). Briefly, 50–100 µl of plasma was diluted with 1x PBS (pH = 7.4) in a 1:7 ratio and applied to the protein G plate. The plate was then washed with 1x PBS (pH = 7.4) in order to remove unbound proteins. Purified IgG was eluted with 0.1 M formic acid (Merck, Darmstadt, Germany) to a final volume of 1 ml and neutralized with 1 M ammonium bicarbonate (Merck, Darmstadt, Germany). Purified IgG was dried in a vacuum centrifuge. IgG N-glycan release and purification for samples from the longitudinal cohort was done with GlycoWorks RapiFluor-MS N-Glycan Kit obtained from Waters Corporation (United States) as described previously (Deriš et al., 2022a). IgG samples from the cross-sectional cohort were first denatured with 1.33% SDS sodium dodecyl sulfate (Invitrogen, Carlsbad, CA) and deglycosylated with PNGase F (ProZyme) overnight at 37°C. Released N-glycans were labeled with 2-aminobenzamide (Sigma-Aldrich) as described previously (Freidin et al., 2016). All labeled N-glycans were then purified by hydrophilic interaction chromatography solid phase extraction (HILIC-SPE). Both RapiFlour-MS labeled IgG N-glycans and 2-aminobenzamide labeled IgG N-glycans were analyzed using ultra-high-performance liquid chromatography based on hydrophilic interactions with fluorescence detection (HILIC-UHPLC-FLD) on Waters Acquity UPLC H-class instruments as described previously (Freidin et al., 2016; Deriš et al., 2022a). Obtained chromatograms of RapiFlour-MS labeled IgG N-glycans were separated into 22 peaks by automated integration (Agakova et al., 2017), while chromatograms of 2-aminobenzamide labeled IgG N-glycans were separated into 24 peaks using a traditional integration algorithm and manual correction (Supplementary Figure S1).

### Glycan clock estimation

Total area normalization was applied to the area under the chromatogram peaks, followed by log-transformation and batch correction using the ComBat method as implemented in R package “sva” (version 3.30.1) (Leek et al., 2012). The glycan peak values were transformed back to the original scale prior to glycan clock estimation.

Glycan clock values were calculated according to Krištić et al., 2014 (Figure 1). First, clock model coefficients were estimated in the female samples in cross-sectional and longitudinal cohorts separately by including only one twin from each twin pair. For the cross-sectional cohort, glycan clock values were calculated using the following formula: 53.83 + (5.24 × GP6) − (0.29 × GP6^2^) − (1.57 × GP14) + (1.76 × GP15). Taking into the consideration the difference in glycan peak annotation due to the difference in peak integration for the longitudinal cohort, formula for the glycan clock was as follows: 50.29 + (5.11 x GP4) − (0.32 x GP4^2^) − (1.24 x GP12) + (1.49 x GP13). Data preprocessing and analysis were performed in R software (version 3.5.1.) (R Core Team, 2018).

### Heritability analysis

Heritability of the glycan clock was estimated using the structural equation modeling (SEM) to decompose the observed phenotypic variance into three latent sources of variation: A—additive genetic variance, C—shared/common environment variance and E—unique environment variance (Neale and Cardon, 1992). Taking into account that the heritability studies require twin pairs, all participants without a co-twin were excluded thus leaving a total of 3,344 females separated into 479 MZ and 1193 DZ twin pairs in the cross-sectional cohort. The same thing was done for the measurements in the longitudinal cohort leaving a total of 549 monozygotic and 1,201 dizygotic twin pairs. Additive genetic effects (A) represent the cumulative impact of genes and they are indicated when the intrapair phenotypic correlation for monozygotic twins (rMZ) is greater than the intrapair phenotypic correlation for dizygotic twins (rDZ). Shared environmental effects (C) result from influences to which both members of a twin pair are exposed regardless of zygosity and contribute equally to rMZ and rDZ, thus, increasing twin similarity. Unique environmental effects (E) are events occurring to one twin but not the other and serve to decrease twin similarity. Unique environmental effects also include the measurement error. Utilizing listed factors (A, C and E), the best model was determined (Figure 1; Supplementary Tables S1, S2). That was done by sequential removal of each factor from the full model (ACE) followed by the likelihood ratio test (*p* = 0.05) to check the deterioration in fit of the various nested models. The best fitting model was selected using the Akaike information criterion (AIC). Lastly, the heritability of the glycan clock was estimated using the most parsimonious model as a proportion of the observed phenotypic variation attributable to genetic factors. Heritability analyses were performed using the package METs (version 1.2.7.1) (Scheike et al., 2014) in R (version 4.0.2).

## Results

**FIGURE 2:**
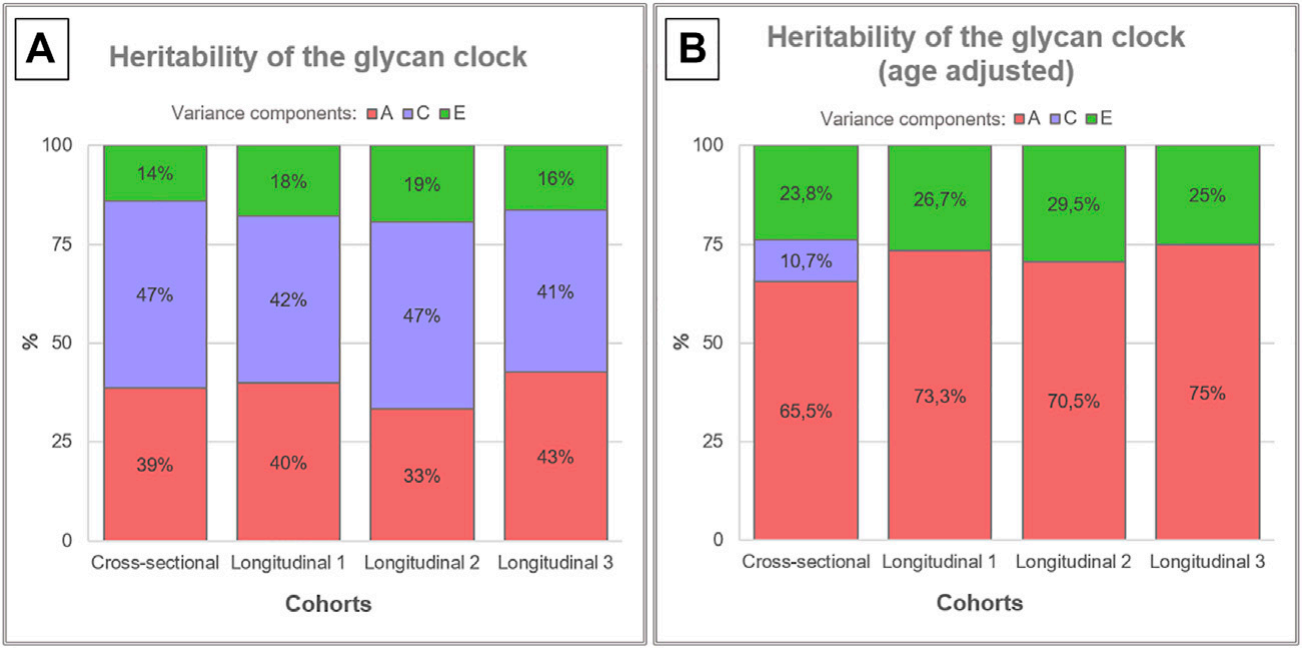
Heritability analysis of the glycan clock. Heritability of the glycan clock was calculated for the cross-sectional and longitudinal cohort with three time points. **(A)** The best fitting model was the full ACE model that includes additive genetic (A), shared environmental (C) and unique environmental (E) variance. **(B)** After correction for the age of the participants the full model for the longitudinal cohort was reduced to AE model.

Glycan clock heritability was estimated in a sample of 3,344 female adult twins from the TwinsUK registry that were a part of one cross-sectional and one longitudinal cohort with three time points. Blood samples from 479 MZ and 1193 DZ twin pairs in the cross-sectional cohort and blood samples from 549 MZ and 1201 DZ twin pairs in the longitudinal cohort were used for the analysis of IgG glycome composition (Figure 1). Representative IgG glycan chromatograms are given in Supplementary Figure S1. Mean time difference was 7.5 years for points 1 and 2 (sd = 3.27) and 6 years for points 2 and 3 (sd = 2.5) in the longitudinal cohort. Glycan clock was estimated from the released IgG glycans and used to calculate heritability. The best fitting model for both cohorts was the full ACE model encompassing genetic (A), shared environmental (C) and unique environmental (E) components (Supplementary Table S1). Heritability estimation for the glycan clock in the cross-sectional study was 38.6%, which suggests a moderate contribution of additive genetic factors to this biomarker of aging (Figure 2A; Supplementary Table S2). The heritability of the glycan clock in the longitudinal cohort was 40%, 33%, and 43% for the first, second and third time point, respectively. The contribution of the environmental factors fell largely on shared environmental variance. The shared environmental influence on the glycan clock variation was 44.6% on average with a minor deviation between the cohorts and different time points. The smallest contribution came from unique environmental factors with an average of 16.8% (Figure 2A).

To better understand the impact of aging on heritability estimates of the glycan clock we performed the same analysis including the age of the participants as a covariate (Supplementary Table S3). This intervention largely inflated the additive genetic influences to an average of 65.5% in the cross-sectional cohort using ACE modeling (Figure 2B). The same phenomenon occurred in the longitudinal cohort but the best fitting model was AE (Supplementary Table S4) with an average genetic contribution of 72.9%. When we compared the results of both cohorts using ACE models, we observed very similar heritability estimates across all datasets averaging at 65.9%. Estimates of shared environmental influence dropped to 10.7%, and unique environmental contribution increased to 23.8% in the cross-sectional cohort. The increase of the unique environmental component was also observed in the longitudinal cohort with all three time points averaging at 27.1% with minor deviations (Figure 2B).

## Discussion

The glycan clock of age, based entirely on IgG N-glycans, can predict biological age with high accuracy (Krištić et al., 2014). To better understand genetic and environmental factors influencing the glycan clock variation, we performed a heritability analysis on the data from two cohorts included in the TwinsUK registry (Figure 1). This was the largest dataset for IgG glycan heritability analysis up to date which gave us the statistical power to detect the heritability of the glycan clock with 95% probability and enabled us to replicate the results to boost the reliability of the data. However, it is important to highlight that heritability studies do come with certain limitations, which can decrease the accuracy of acquired data. They are inherently restricted because all estimates heavily depend on environmental variance. Moreover, twin design assumes that correlations and interactions of genes and environment are minimal and cannot take the effect of epigenetics into account. All of these factors can falsely attribute the contribution of environmental factors to genetics leading to artificially inflated heritability estimates. However, classic heritability studies currently offer the best approximation of environmental and genetic contributions to the analyzed phenotype (Rijsdijk and Sham, 2002; Kaminsky et al., 2009; Manolio et al., 2009; Mayhew and Meyre, 2017).

Previous heritability studies conducted on IgG glycans demonstrated that IgG glycome has a variable heritability depending on the exact glycan structure analyzed. Most of the glycan traits turned out to be at least 50% heritable, with only a few having a low genetic contribution (Pučić et al., 2011; Menni et al., 2013). This tight genetic control was later explained by complex gene networks discovered through numerous GWA (genome-wide association) studies conducted on IgG glycan traits (Huffman et al., 2011; Lauc et al., 2013; Shen et al., 2017; Wahl et al., 2018b; Klarić et al., 2020). One of the first GWA studies on IgG glycan phenotype revealed that IgG glycosylation is not only regulated through the expression of glycosyltransferases that add specific sugars to the growing IgG glycan but through different genes with a previously unknown role in IgG glycosylation (Lauc et al., 2013). A recent GWAS discovered a gene network involving 27 loci implicated in the process of IgG glycosylation with most of them associated with various autoimmune and inflammatory conditions (Klarić et al., 2020). Furthermore, part of this gene network was functionally validated *in vitro* utilizing a recently developed transient expression system based on CRISPR tools (Mijakovac et al., 2021, 2022). Despite this tight genetic control of the IgG glycome that is currently being unraveled, heritability analysis of the glycan clock revealed only a moderate genetic contribution averaging around 39% for both cohorts. In the longitudinal cohort, IgG glycome composition changed substantially due to aging but the heritability estimates remained fixed. The contribution of the environmental factors fell largely on shared environmental variance with only a proportion coming from the unique environment.

Considering the fact that aging has the largest impact on IgG glycosylation, we included the age of the individuals as a covariate in the heritability analysis of the glycan clock. This intervention largely deflated the contribution of shared environmental variance to the point where AE model became the best fitting one for the longitudinal cohort. As a consequence, the heritability estimates almost doubled to an average of 71%. The observed increase in the genetic component could be a consequence of chronological age as a shared environmental variance characteristic for every individual and determined by their genetic makeup and epigenetic regulation. Aging in general leads to epigenetic mediated deregulation of genes so it is safe to assume that the glycan clock heritability estimates are not devoid of this effect (Saldanha and Watanabe, 2015). Different studies reported that the process of protein glycosylation is under strong epigenetic control (Horvat et al., 2013; Agrawal et al., 2014; Greville et al., 2016, 2021; Indellicato and Trinchera, 2021). We hypothesize that aging related epigenetic changes such as altered DNA methylation, abnormal chromatin state, altered histone modifications and deregulation of non-coding RNAs (Saldanha and Watanabe, 2015) also impact the glycan clock variation which is reflected in the inflated heritability estimates after age correction.

The glycan clock is a very powerful predictor of chronological and biological age but more importantly, it can be turned by simple lifestyle changes as many recent studies have reported. Lifestyle decisions such as exercise and nutrition can have an immense impact on biological age measured by the glycan clock (Peng et al., 2019; Tijardović et al., 2019; Greto et al., 2021). But how much can the glycan clock actually be turned remains an open question. We argue that the contribution of the unique environmental variance to the phenotypic variation of the glycan clock is comparable to the contribution of lifestyle decisions. After we corrected the data for the age of the individuals, estimates for the unique environmental variance averaged at 26%. This result emphasizes the high plasticity of the IgG glycome in response to environmental stimuli and in part supports the notion that the glycan clock can be rejuvenated by simple lifestyle choices. Mechanisms by which the lifestyle choices affect the glycan clock are still unknown, but most of the recent studies point to the epigenetic regulation of IgG glycosylation with an accent on DNA methylation (Menni et al., 2013; Lauc et al., 2014; Wahl et al., 2018a; Klasić and Zoldoš, 2021).

**FIGURE 3:**
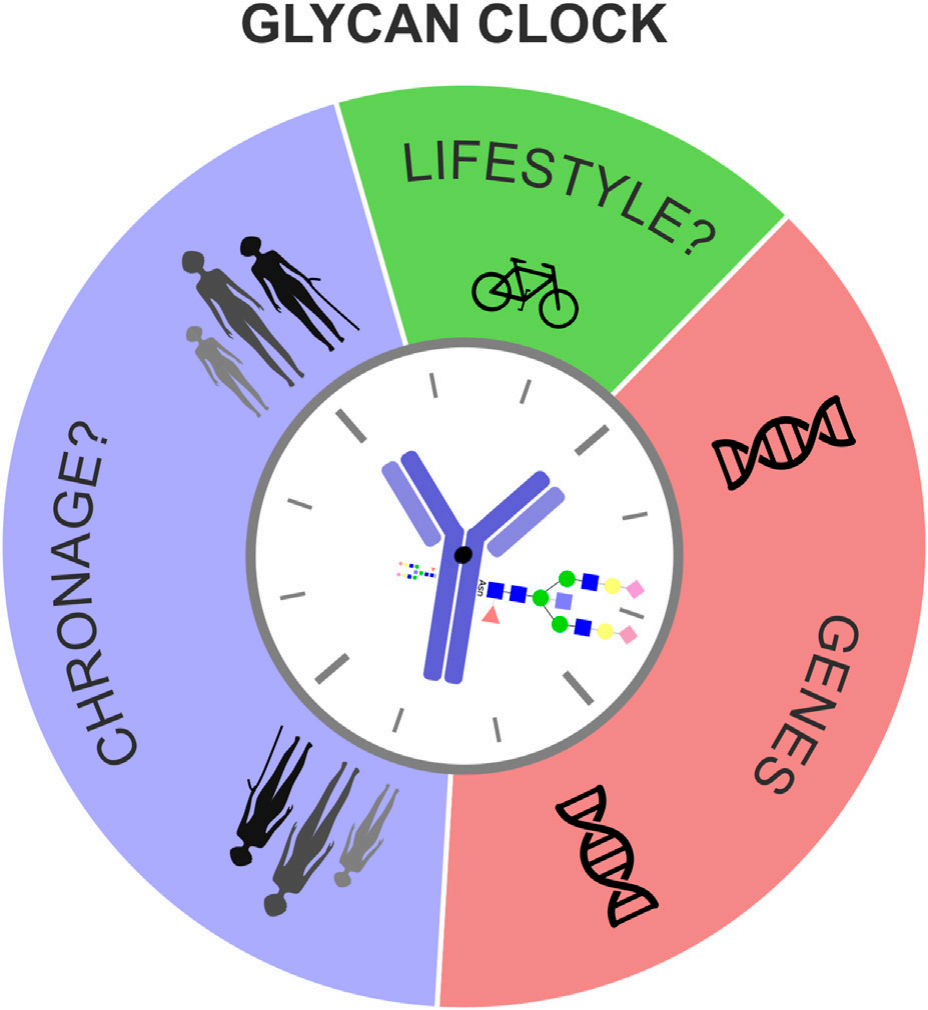
Genes, chronological age and lifestyle influence biological age measured by the glycan clock. Biological age, measured by the glycan clock is determined by a complex interplay between genetic and environmental factors. Even though the genetic template partly explains the phenotypic variation of the glycan clock this heritability analysis showed how big of a role environment has. Chronological age (ChronAge), that causes biological fatigue, together with fixed genetic information and unique lifestyle choices dictates the glycan clock ticking.

In conclusion, we propose that the biological age, measured by the glycan clock, is determined by a complex interplay of fixed genetic information, chronological age (ChronAge) of the individual and unique lifestyle choices, mediated by the plasticity of the human epigenome (Figure 3). Responsiveness of the glycan clock to a healthier lifestyle and its potential to integrate genetic, epigenetic and environmental cues puts this biomarker as one of the most alluring predictors of biological age in modern personalized medicine.

## Data Availability

The original contributions presented in the study are included in the article/Supplementary Material, further inquiries can be directed to the corresponding author.
