# Supplementary figures and images for: Heritability of the glycan clock of biological age
Source: Front Cell Dev Biol. 2022 Dec 22;10:982609. doi: 10.3389/fcell.2022.982609 (PMC9815111; doi:10.3389/fcell.2022.982609)

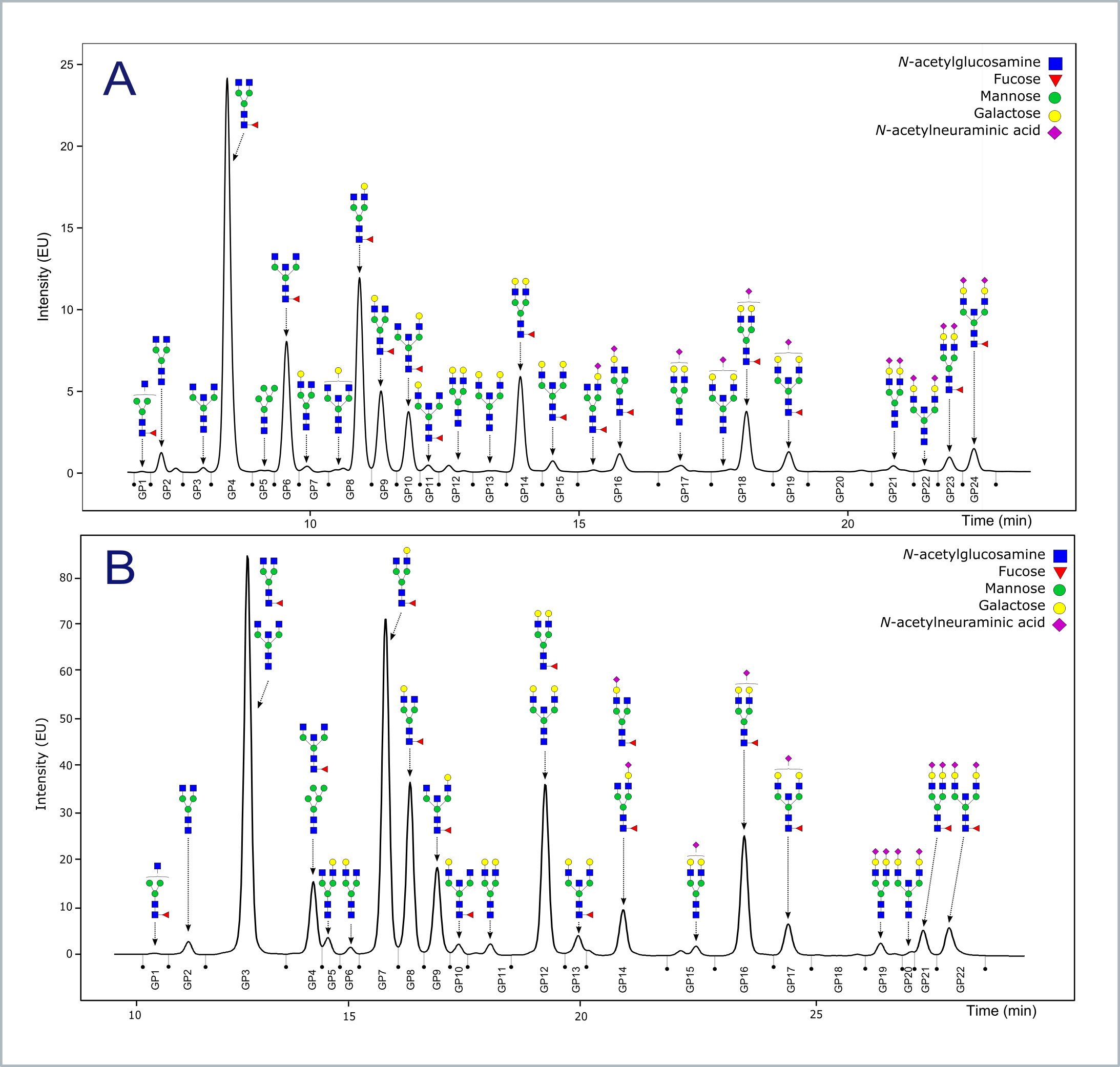

Supplement: Supplementary file 1 [file Image1.TIFF]
