# Supplementary material for: Heritability of the glycan clock of biological age
Source: Front Cell Dev Biol. 2022 Dec 22;10:982609. doi: 10.3389/fcell.2022.982609 (PMC9815111; doi:10.3389/fcell.2022.982609)
Supplement: Supplementary file 3 [file DataSheet1.docx]

**Supplementary figure 1.** Representative chromatographic profiles of the IgG N-glycome. **[A]** HILIC-UHPLC-FLD chromatographic profile of the IgG 2-aminobenzamide labeled N-glycome separated into 24 peaks (cross-sectional cohort). **[B]** HILIC-UHPLC-FLD chromatographic profile of the IgG RapiFluor-MS labeled N-glycome separated into 22 peaks (longitudinal cohort).

**Supplementary Table 1.** Twin correlations for the saturated model and the most parsimonious model. Based on these correlation values, the ACE model was fitted in all the heritability analyses.

**Supplementary table 2.** Summary of the heritability estimates for all analysed traits.

**Supplementary table 3.** Summary of the heritability estimates for all analysed traits. Including age adjustment.

**Supplementary table 4.** Twin correlations for the saturated model and the most parsimonious model. Based on these correlation values, the ACE model was fitted in all the heritability analyses. Corrected by age.
